# The Electronic Structure of Planar Rhombic Co_2_O_2_

**DOI:** 10.1021/acs.jpca.5c06695

**Published:** 2026-01-12

**Authors:** Dou Du, Namin Xiao, Xingwu Li, Maria Dimitrova, Dage Sundholm, Xiao-Gen Xiong

**Affiliations:** † 74688 AECC Beijing Institute of Aeronautical Materials, Beijing 100095, China; ‡ 3835 University of Helsinki, Department of Chemistry, P.O. Box 55 (A.I. Virtanens plats 1), Helsinki FIN-00014, Finland; § Sino-French Institute of Nuclear Engineering and Technology, 26469 Sun Yat-sen University, Zhuhai 519082, P. R. China; ∥ CNPRI-SYSU Joint Research Center for Coolant Chemistry of Nuclear Reactor, Zhuhai 519082, P. R. China

## Abstract

Low-lying valence states of the planar rhombic (*D*_2h_) structure of Co_2_O_2_ have been computationally studied at density functional theory (DFT) levels, at the singles and doubles coupled-cluster level augmented with a perturbative treatment of the triples (CCSD­(T)), at the complete active space self-consistent field (CASSCF) level, and at the second-order complete active space perturbation theory (CASPT2) level. Calculations at the DFT, CCSD­(T), and CASSCF levels suggest that the ground state is a high-spin septet state, whereas the CASPT2 calculations yield a singlet ground state (^1^ *A*_*g*_). The wave function of the CCSD­(T) ground state (^7^ *B*_2*u*_) is dominated by one Slater determinant and is therefore well described using single-reference methods, whereas the configuration interaction coefficient (CI) of the reference determinant (*C*_0_) of the ^1^ *A*_*g*_ state is only 0.519 at the CASSCF level. High-spin states are stabilized at the DFT level by increasing the amount of Hartree–Fock (HF) exchange in the functional. Static and dynamic correlation effects must be considered to obtain the correct ground state of Co_2_O_2_. DFT calculations of the magnetically induced current density (MICD) susceptibility of the ^1^ *A*_*g*_ state using the TPSSh functional show that the molecule sustains a strong diatropic ring current, which has significant contributions from both σ and π orbitals.

## Graphical abstract

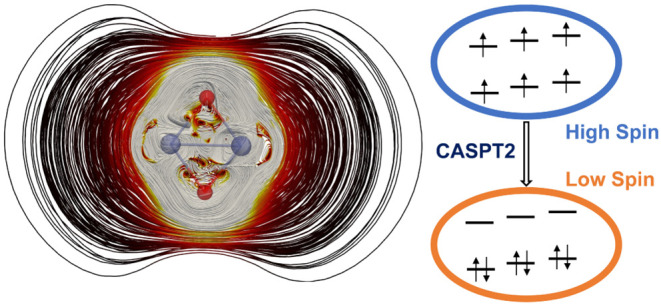

## 1 Introduction

Cobalt (Co) and its oxides (Co_*x*_O_*y*_) are important in industrial and technological applications due to their unique chemical and physical properties. Metallic cobalt is used in the production of high-performance alloys, in rechargeable batteries, and as catalysts, particularly in hydrogen production reactions and Fischer–Tropsch synthesis.^1–4^ Cobalt oxides, such as CoO, Co_2_O_2_, Co_2_O_3_, and Co_2_O_2_ have electronic, magnetic, and catalytic properties that make them useful in energy storage devices^5^ supercapacitors^6,7^ and sensors.^8,9^ Complexes containing Co_*x*_O_*y*_ units serve as efficient electrocatalysts in oxygen evolution and reduction reactions,^10–12^ which are important for fuel cells and water-splitting technologies.

Cobalt-containing compounds have a complex electronic structure due to the partially filled 3*d* shell of the cobalt atom that leads to a high density of near-degenerate molecular electronic valence states. The near degeneracy arises from the large number of possible electron configurations and spin states that cobalt can adopt in its various oxidation states and coordination environments. Therefore, conventional single-reference electronic structure methods often fail to accurately describe the electronic properties of cobalt-containing molecules. To capture the static and dynamic correlation effects of the electrons in the *d* shell, multiconfigurational approaches, such as the complete active space self-consistent field (CASSCF), multireference perturbation theory (MRPT) or multireference configuration interaction (MRCI) calculations are required.^13^

Computational methods that consider both static and dynamic correlation effects were used to calculate accurate spectroscopic properties for low-lying valence states of cobalt monoxide.^14–17^ Accurate results for a large number of valence states of CoO were obtained when performing calculations at *ab initio* correlation levels of theory.^15,18,19^ Electronic structure calculations have also provided information on the electronic properties of cobalt-oxide clusters (Co_*x*_O_*y*_).^20–24^ Since *ab initio* correlation approaches are computationally expensive, density functional theory (DFT) calculations are often used even though they may not accurately account for strong electron-correlation effects in transition-metal compounds with nearly degenerate electronic states.^25^ Researchers have therefore employed DFT-based computational methods beyond the standard DFT approach, such as Hubbard corrected DFT methods (DFT+U).^26^ Such calculations have enabled predictions of oxidation states, spin configurations, and electronic transitions of cobalt-containing compounds, leading to deeper insights into the catalytic activity, magnetic properties, and charge-transport mechanisms of cobalt oxides. Since the computational efficiency continues to grow, further methodological developments, including machine learning-assisted electronic structure methods^27^ are expected to push the limits of the precision and efficiency of studies of these technologically important materials.

The properties of the chemical bonding of cobalt oxides (Co_*x*_O_*y*_) are governed by the interplay between covalent, ionic, and metallic interactions, which are influenced by the oxidation state of the cobalt atom and its local coordination environment. The oxidation state of cobalt is Co­(II) in the solid state of CoO, forming a rock-salt structure where the bonding is mainly ionic due to electron transfer from Co­(II) to oxygen. The Co–O bond is to some extent covalent due to the hybridization between the 3*d* orbitals of Co and the oxygen 2*p* orbitals. Cobalt-oxide clusters such as Co_3_O_4_ and Co_2_O_2_ have mixed-valence states leading to a complex electronic structure and variable oxidation states. The Co–O–Co superexchange interactions contribute to their unique electronic and magnetic properties. In low-dimensional cobalt oxides and in nanostructures, quantum confinement effects modify the chemical bonding, making them efficient in energy applications and as catalysts.

Here, we systematically investigate the electronic structure of Co_2_O_2_ by performing quantum chemistry calculations at *ab initio* correlation and DFT levels of theory. Our work shows the importance of considering both static and dynamic correlation effects when studying low-lying valence states of Co_2_O_2_. The article is outlined as follows. The computational details are given in the next section, and the results are discussed in Section 3 and the main conclusions are drawn in Section 4.

## 2 Computational Methods

We performed density functional theory (DFT) and wave function theory (WFT) calculations on electronic spin states of Co_2_O_2_. The molecular structures of spin states with the spin quantum number *S* = 0, 1, 2, and 3 were optimized at the DFT level using a series of DFT functionals, i.e., BP86,^28,29^ PBE,^30^ B3LYP,^31,32^ PBE0,^33^ TPSS,^34^ TPSSh,^35^ and ωB97X^36^ combined with the def2-TZVPP basis sets^37,38^ using the Molpro2022 program package.^39^ The optimized rhombic structures belong to the *D*_2h_ point group, which was also obtained in previous experimental^24,40^ and computational^41^ studies. The vibrational frequencies were calculated to confirm that the molecular structures are minima on the potential energy surface. The relative energy differences between the lowest spin states of Co_2_O_2_ were determined by performing single-point calculations at coupled cluster and multireference levels of theory using Molpro2022.

Due to the complex electronic structure and the high symmetry of Co_2_O_2_, determining the energetically lowest electron configuration is laborious. Therefore, we adopt the following strategy to find the relative order of the valence states. We began by performing single-point calculations at the state-average complete active space self-consistent field (SA-CASSCF) level,^42–44^ for each spin multiplicity and states belonging to the possible irreducible representations. For example, the SA-CASSCF calculation on the septet (*S* = 3) states considers the eight possible electronic states (^7^ *A*_*g*_, ^7^ *B*_1*g*_, ^7^ *B*_2*g*_, ^7^ *B*_3*g*_, ^7^ *A*_*u*_, ^7^ *B*_1*u*_, ^7^ *B*_2*u*_ and ^7^ *B*_3*u*_) of the *D*_2h_ point group. The energetically lowest electron configuration obtained in the SA-CASSCF calculations was used as the reference determinant in calculations at the coupled-cluster singles and doubles level augmented with a perturbative treatment of the triples level (CCSD­(T)).^45–47^ The molecular structures of the individual states were optimized at the CCSD­(T) level using the augmented all-electron correlation-consistent triple-ζ basis sets (aug-cc-pVTZ).^48,49^ The same strategy was also used to determining the electron configuration of the singlet, triplet, and quintet states studied at the CCSD­(T) level. Although we also performed the SA-CASSCF calculations for singlet states, the subsequent singlet CCSD­(T) calculation was carried out only for the ^1^ *A*_g_ state because single-reference CCSD­(T) calculations cannot be applied to open-shell singlet states.

Static and dynamic correlation effects were simultaneously considered by performing calculations at the state-specific complete active space second-order perturbation theory level (SS-CASPT2)^50,51^ on the selected low-lying states obtained in the CCSD­(T) calculations. The CCSD­(T) optimized geometries for the selected states were used in the multireference calculations.

The active space used in the SA-CASSCF and SS-CASPT2 calculations includes 18 molecular orbitals dominated by the 3*d*/4*s* atomic orbitals of Co and the 2*p* atomic orbitals of O. The number of active electrons in the CASSCF calculations is 26 yielding a CAS (26e,18o) active space. The active orbitals are shown in Figure 1.

**Figure 1:**
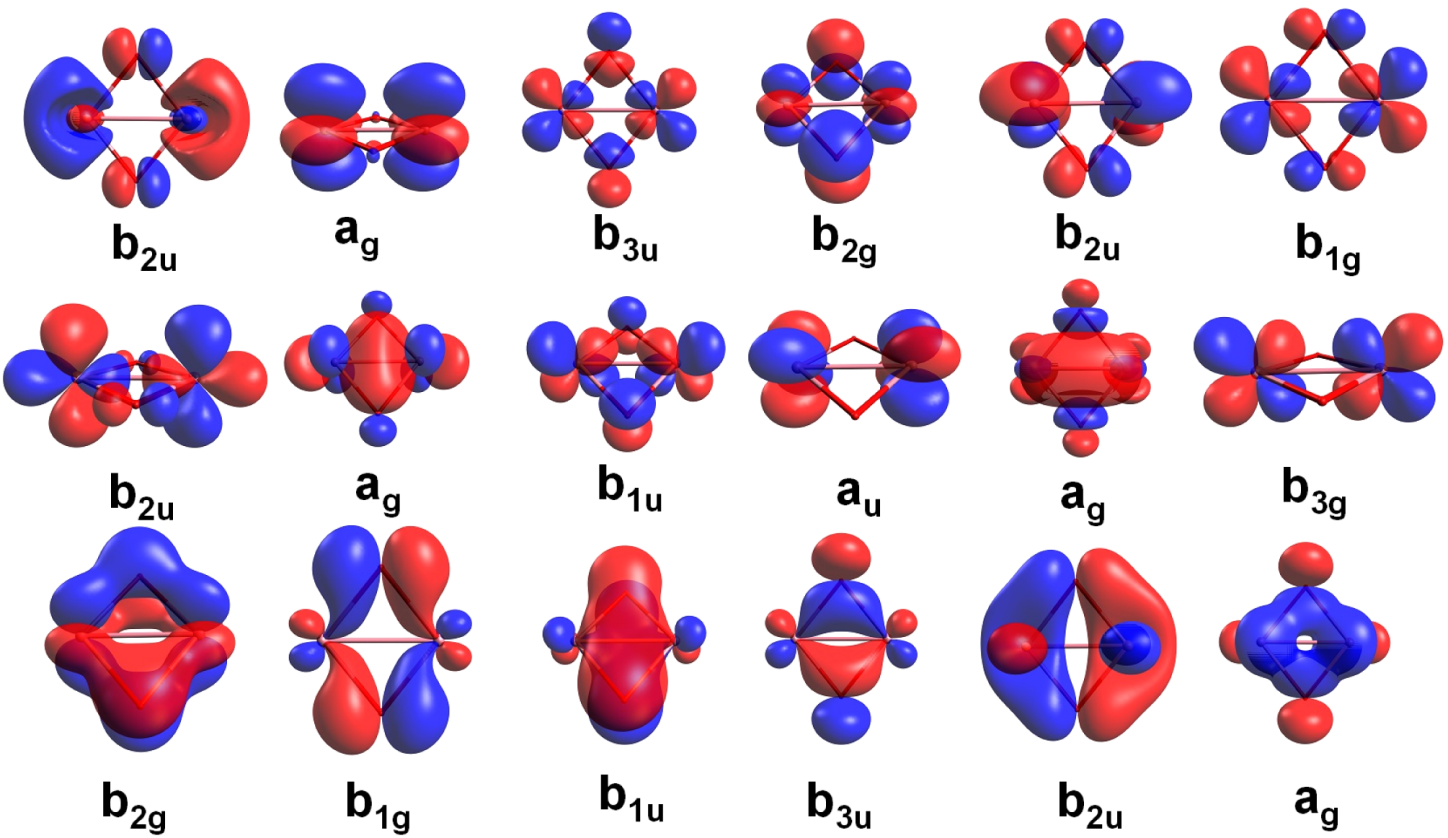
Active orbitals in the CASSCF calculations on Co_2_O_2_ (isocontour = 0.05 au).

Basis-sets effects were investigated by performing CASPT2/aug-cc-pVQZ-t calculations, where t denotes truncated. We removed the basis functions with the highest angular momentum, that is, *h*-functions of Co and *g*-functions of O, from the original aug-cc-pVQZ basis sets to make the demanding CASPT2 calculations feasible. The CASPT2/aug-cc-pVQZ-t calculations were performed with the ORZ package^52^ because these calculations were not feasible with Molpro2022 on our workstations.

The magnetically induced current density (MICD) susceptibility was calculated at the DFT level using the gauge-including magnetically induced currents (GIMIC) method.^53–56^ The input data for the GIMIC calculations were obtained by performing nuclear magnetic shielding calculations with Turbomole.^57–60^ In the MICD calculations, we used the TPSSh functional^34,35^ and large polarization-consistent (pcseg-3-t) basis sets^61,62^ to ensure that the charge conservation condition of the MICD susceptibility is nearly met also near the Co atoms. The letter t in the basis-set name means that the *g*- and *h*-type basis functions were omitted. The type 5 integration grid as implemented in Turbomole was used.^63^

## 3 Results and Discussions

### 3.1 DFT Calculations

Previous DFT studies yielded different ground states of Co_2_O_2_ depending on the employed functional and the computational approach. Chertihin et al. suggested that ^7^ *A*_*u*_ is the ground state based on B3LYP calculations, while the ^5^ *B*_1*g*_ and ^5^ *B*_3*u*_ states were 27 and 7 kcal mol^–1^ higher in energy, respectively.^24^ Gutsev et al. used the BPW91 functional and concluded that the ground state is a singlet.^41^ Broken-symmetry DFT calculations using the TPSSh functional by Staemmler et al. suggested that Co_2_O_2_ has a singlet ground state.^21^ Uzunova and Mikosch obtained singlet ground state at the DFT level using the B1LYP functional.^22^

We calculated the total energy of the fully optimized molecular structure of selected states using various density functional approximations (DFA) because the energetic order of the various states depends on the chosen functional. The relative energies are given in Table 1, where one can see that the ground state is ^5^ *B*_2*g*_ when using the BP86 and PBE functionals at the generalized gradient approximation (GGA). The calculations using the B3LYP and PBE0 hybrid functionals suggest that ^7^ *B*_2*u*_ is the ground state.

**Table 1:** Relative Energies of the Lowest Valence States Calculated at DFT Levels of Theory^a^

State	BP86	PBE	B3LYP	PBE0	TPSS	TPSSh	ωB97X
^1^ *A*_*g*_	8.25	7.56	65.65	83.54	12.69	38.38	81.25
^3^ *A*_*g*_	8.37	7.82	62.91	80.85	15.87	37.14	84.55
^3^ *B*_1*u*_	4.78	4.23	61.83	80.07	9.06	35.22	82.99
^3^ *B*_2*u*_	24.07	23.84	55.81	70.98	26.41	37.67	70.72
^5^ *A*_*g*_	20.27	20.28	22.01	28.26	17.47	19.67	28.25
^5^ *B*_1*g*_	6.85	6.07	55.81	71.29	10.95	33.63	77.74
^5^ *B*_2*g*_	0.00	0.00	25.92	36.20	4.64	11.85	39.67
^5^ *B*_1*u*_	11.26	10.98	39.74	51.61	17.09	25.43	55.53
^7^ *A*_*g*_	14.77	14.62	20.02	23.77	20.82	16.79	26.38
^7^ *B*_3*g*_	33.49	33.42	41.35	49.14	34.31	35.72	55.46
^7^ *A*_*u*_	3.25	3.59	6.89	10.54	0.00	3.92	12.43
^7^ *B*_1*u*_	10.76	10.65	18.15	21.89	17.13	13.29	24.48
^7^ *B*_2*u*_	17.72	18.10	0.00	0.00	9.21	0.00	0.00

a The def2-TZVPP basis sets were used. The energies are given in kcal mol^–1^ .

Since the electronic energies calculated at the GGA level for ^5^ *B*_2*g*_ and ^7^ *A*_*u*_ are very close, zero-point energy (ZPE) corrections can change their order. Considering the ZPE corrections at the PBE level does not change the order of the lowest states because the difference between the ZPE corrections of ^5^ *B*_2*g*_ (4.05 kcal mol^–1^) and ^7^ *A*_*u*_ (3.74 kcal mol^–1^) is only 0.31 kcal mol^–1^.

At the meta-GGA (TPSS) level, the high-spin ^7^ *A*_*u*_ state is the ground state. DFT calculations using the TPSSh functional having 10% HF exchange added to the TPSS functional suggest that ^7^ *B*_2*u*_ is the ground state. The difference between the ZPE corrections of 0.33 kcal mol^–1^ calculated at the PBE level for ^7^ *A*_*u*_ (3.74 kcal mol^–1^) and ^7^ *B*_2*u*_ (3.41 kcal mol^–1^) is too small to affect the order of the lowest states. The PBE0 hybrid functional and the ωB97X range-separated hybrid functional also suggest that ^7^ *B*_2*u*_ is the ground state. Thus, including the exact exchange into the functional increases the stability of the high-spin states.

Calculations at the TPSSh level without any symmetry constraints suggest that the quintet state is the ground state. Scalar relativistic calculations at the TPSSh level yield almost the same relative energies and the same order of the states as obtained at the nonrelativistic level. Considering spin–orbit effects at the exact two-component (X2C-2c) relativistic level^58,64^ leads to a low-spin state $\left(\left\langle S_{x}\right\rangle =\left\langle S_{y}\right\rangle =\left\langle S_{z}\right\rangle =0\right)$ with significant spin contamination (⟨*S* ^2^⟩ = 2.68). The singlet state is the ground state according to broken-symmetry (BS) calculations at the TPSS level using the BS approach in Gaussian.^65^ In the BS calculations, the triplet, quintet, and the septet states are energetically 9.77, 5.33, and 6.07 kcal mol^–1^ above the singlet, respectively.

The TPSSh calculations without symmetry constraints yielded symmetry-broken molecular structures (*C*_s_) for the triplet and quintet states. The Co–O distances are 1.809, 1.784 (average), 1.794 (average), and 1.823 Å for the singlet to the septet state, respectively. The Co–O–Co angles are 80.1, 82.0, 79.4, and 79.1°, respectively. The bond lengths are slightly longer than the experimental value of 1.765 ± 0.01 Å and the angle is also smaller than the experimental value of 87.5°.^40^ Population analysis based on occupation numbers suggests a Co charge of +0.8*e* for the four states, whereas Staemmler et al., reported a Mulliken charge of +2*e* for Co.

### 3.2 CCSD­(T) Calculations

The CCSD­(T) calculations suggest that ^7^ *B*_2*u*_ is the ground state of Co_2_O_2_. However, it is only 0.19 kcal mol^–1^ below the ^7^ *A*_*u*_ state. The ZPE of ^7^ *A*_*u*_ and ^7^ *B*_2*u*_ calculated at the PBE level are 3.74 kcal mol^–1^ and 3.41 kcal mol^–1^, respectively. The ZPE correction calculated at the PBE level increases the energy difference between the two states. Thus, ^7^ *B*_2*u*_ is the ground state at the CCSD­(T) level when considering the ZPE corrections at the PBE level. The spin–orbit splittings of the *a* ^4^ *F* ground state of Co^2+^ are 841.2 (2.405), 610.1 (1.744), and 415.5 (1.188) cm^–1^ (kcal mol^–1^)^66^ introducing additional uncertainties on the order of almost degenerate states.

The singlet state (^1^ *A*_*g*_) is 16.10 kcal mol^–1^ above the ^7^ *B*_2*u*_ state. ^3^ *B*_1*u*_ and ^3^ *B*_3*u*_ are the most stable triplet states lying 22.09 kcal mol^–1^ and 21.92 kcal mol^–1^ above the ^7^ *B*_2*u*_ state, respectively. ^5^ *B*_2*g*_ is the most stable quintet state, which is 6.78 kcal mol^–1^ above ^7^ *B*_2*u*_. The CCSD (T) energies of the lowest valence states in Table 2 show that some of the high-spin states are energetically below the lowest singlet state.

**Table 2:** Electron Configuration of the Reference Determinant of the CCSD­(T) Calculations^a^

State	Electronic configuration	*E*_total_	Δ*E*	*D*_1_	*T*_1_
^1^ *A*_*g*_	(10*a*_*g*_)^2^(5*b*_3*u*_)^2^(7*b*_2*u*_)^2^(8*b*_2*u*_)^0^(4*b*_1*g*_)^2^(4*b*_1*u*_)^2^(1*b*_2*g*_)^2^(3*b*_3*g*_)^2^(1*a*_*u*_)^2^	–2914.542455	16.10	0.173	0.033
^3^ *A*_*g*_	(10*a*_*g*_)^2^(5*b*_3*u*_)^2^(7*b*_2*u*_)^1^(8*b*_2*u*_)^1^(4*b*_1*g*_)^2^(4*b*_1*u*_)^2^(1*b*_2*g*_)^2^(3*b*_3*g*_)^2^(1*a*_*u*_)^2^	–2914.533989	21.41	0.155	0.028
^3^ *B*_1*g*_	(10*a*_*g*_)^2^(5*b*_3*u*_)^2^(7*b*_2*u*_)^2^(8*b*_2*u*_)^0^(4*b*_1*g*_)^2^(4*b*_1*u*_)^2^(2*b* ^2*g*^)^1^(3*b*_3*g*_)^1^(1*a*_*u*_)^2^	–2914.522180	28.82	0.166	0.034
^3^ *B*_2*g*_	(10*a*_*g*_)^2^(5*b*_3*u*_)^2^(7*b*_2*u*_)^2^(8*b*_2*u*_)^2^(4*b*_1*g*_)^1^(4*b*_1*u*_)^2^(2*b*_2*g*_)^2^(2*b*_3*g*_)^1^(1*a*_*u*_)^2^	–2914.515600	32.95	0.232	0.047
^3^ *B*_3*g*_	(10*a*_*g*_)^2^(5*b*_3*u*_)^2^(7*b*_2*u*_)^2^(8*b*_2*u*_)^1^(4*b*_1*g*_)^2^(4*b*_1*u*_)^1^(1*b*_2*g*_)^2^(3*b*_3*g*_)^2^(1*a*_*u*_)^2^	–2914.512389	34.96	0.165	0.035
^3^ *A*_*u*_	(10*a*_*g*_)^2^(5*b*_3*u*_)^2^(7*b*_2*u*_)^2^(8*b*_2*u*_)^1^(4*b*_1*g*_)^2^(4*b*_1*u*_)^2^(2*b* ^2*g*^)^1^(2*b*_3*g*_)^2^(1*a*_*u*_)^2^	–2914.495150	45.78	0.165	0.035
^3^ *B*_1*u*_	(10*a*_*g*_)^2^(5*b*_3*u*_)^2^(7*b*_2*u*_)^2^(8*b*_2*u*_)^1^(4*b*_1*g*_)^2^(4*b*_1*u*_)^2^(1*b*_2*g*_)^2^(3*b*_3*g*_)^1^(1*a*_*u*_)^2^	–2914.532901	22.09	0.154	0.033
^3^ *B*_2*u*_	(10*a*_*g*_)^2^(5*b*_3*u*_)^2^(7*b*_2*u*_)^2^(8*b*_2*u*_)^2^(4*b*_1*g*_)^2^(4*b*_1*u*_)^1^(1*b*_2*g*_)^2^(3*b*_3*g*_)^1^(1*a*_*u*_)^2^	–2914.529622	24.15	0.259	0.043
^3^ *B*_3*u*_	(10*a*_*g*_)^2^(5*b*_3*u*_)^2^(7*b*_2*u*_)^2^(8*b*_2*u*_)^1^(4*b*_1*g*_)^1^(4*b*_1*u*_)^2^(1*b*_2*g*_)^2^(3*b*_3*g*_)^2^(1*a*_*u*_)^2^	–2914.533170	21.92	0.167	0.044
^5^ *A*_*g*_	(10*a*_*g*_)^2^(5*b*_3*u*_)^2^(7*b*_2*u*_)^2^(8*b*_2*u*_)^2^(4*b*_1*g*_)^2^(4*b*_1*u*_)^1^(2*b* ^2*g*^)^1^(3*b*_3*g*_)^1^(1*a*_*u*_)^1^	–2914.546997	13.24	0.204	0.039
^5^ *B*_1*g*_	(10*a*_*g*_)^2^(5*b*_3*u*_)^2^(7*b*_2*u*_)^1^(8*b*_2*u*_)^1^(4*b*_1*g*_)^2^(4*b*_1*u*_)^2^(2*b* ^2*g*^)^1^(3*b*_3*g*_)^1^(1*a*_*u*_)^2^	–2914.535356	20.55	0.138	0.027
^5^ *B*_2*g*_	(10*a*_*g*_)^2^(5*b*_3*u*_)^2^(7*b*_2*u*_)^2^(8*b*_2*u*_)^1^(4*b*_1*g*_)^2^(4*b*_1*u*_)^1^(2*b* ^2*g*^)^1^(3*b*_3*g*_)^1^(1*a*_*u*_)^2^	–2914.557303	6.78	0.150	0.033
^5^ *B*_3*g*_	(10*a*_*g*_)^2^(5*b*_3*u*_)^2^(7*b*_2*u*_)^2^(8*b*_2*u*_)^1^(4*b*_1*g*_)^2^(4*b*_1*u*_)^2^(2*b* ^2*g*^)^1^(3*b*_3*g*_)^1^(1*a*_*u*_)^1^	–2914.546135	13.79	0.161	0.036
^5^ *A*_*u*_	(10*a*_*g*_)^2^(5*b*_3*u*_)^2^(7*b*_2*u*_)^2^(8*b*_2*u*_)^1^(4*b*_1*g*_)^2^(4*b*_1*u*_)^1^(2*b*_2*g*_)^2^(3*b*_3*g*_)^1^(1*a*_*u*_)^1^	–2914.503480	40.55	0.144	0.034
^5^ *B*_1*u*_	(10*a*_*g*_)^2^(5*b*_3*u*_)^2^(7*b*_2*u*_)^2^(8*b*_2*u*_)^1^(4*b*_1*g*_)^2^(4*b*_1*u*_)^1^(2*b* ^2*g*^)^1^(3*b*_3*g*_)^2^(1*a*_*u*_)^1^	–2914.532410	22.40	0.163	0.037
^5^ *B*_2*u*_	(10*a*_*g*_)^2^(5*b*_3*u*_)^2^(7*b*_2*u*_)^1^(8*b*_2*u*_)^1^(4*b*_1*g*_)^2^(4*b*_1*u*_)^2^(2*b* ^2*g*^)^1^(3*b*_3*g*_)^2^(1*a*_*u*_)^1^	–2914.538782	18.40	0.173	0.034
^5^ *B*_3*u*_	(10*a*_*g*_)^2^(6*b*_3*u*_)^2^(7*b*_2*u*_)^1^(8*b*_2*u*_)^1^(4*b*_1*g*_)^2^(4*b*_1*u*_)^1^(2*b* ^2*g*^)^1^(3*b*_3*g*_)^2^(1*a*_*u*_)^0^	–2914.455984	70.36	0.298	0.058
^7^ *A*_*g*_	(10*a*_*g*_)^2^(6*b*_3*u*_)^1^(7*b*_2*u*_)^1^(8*b*_2*u*_)^1^(4*b*_1*g*_)^1^(4*b*_1*u*_)^2^(2*b* ^2*g*^)^1^(3*b*_3*g*_)^2^(1*a*_*u*_)^1^	–2914.548003	12.61	0.180	0.042
^7^ *B*_1*g*_	(10*a*_*g*_)^2^(6*b*_3*u*_)^1^(7*b*_2*u*_)^1^(8*b*_2*u*_)^1^(4*b*_1*g*_)^1^(4*b*_1*u*_)^1^(2*b* ^2*g*^)^1^(3*b*_3*g*_)^2^(1*a*_*u*_)^2^	–2914.513148	34.49	0.502	0.085
^7^ *B*_2*g*_	(10*a*_*g*_)^1^(6*b*_3*u*_)^1^(7*b*_2*u*_)^2^(8*b*_2*u*_)^2^(4*b*_1*g*_)^1^(4*b*_1*u*_)^1^(2*b* ^2*g*^)^1^(3*b*_3*g*_)^1^(1*a*_*u*_)^2^	–2914.535109	20.70	0.278	0.050
^7^ *B*_3*g*_	(10*a*_*g*_)^1^(6*b*_3*u*_)^1^(7*b*_2*u*_)^2^(8*b*_2*u*_)^2^(4*b*_1*g*_)^1^(4*b*_1*u*_)^2^(2*b* ^2*g*^)^1^(3*b*_3*g*_)^1^(1*a*_*u*_)^1^	–2914.542031	16.36	0.379	0.061
^7^ *A*_*u*_	(10*a*_*g*_)^2^(6*b*_3*u*_)^1^(7*b*_2*u*_)^2^(8*b*_2*u*_)^1^(4*b*_1*g*_)^1^(4*b*_1*u*_)^1^(2*b* ^2*g*^)^1^(3*b*_3*g*_)^1^(1*a*_*u*_)^2^	–2914.567800	0.19	0.231	0.046
^7^ *B*_1*u*_	(10*a*_*g*_)^2^(6*b*_3*u*_)^1^(7*b*_2*u*_)^2^(8*b*_2*u*_)^1^(4*b*_1*g*_)^1^(4*b*_1*u*_)^2^(2*b* ^2*g*^)^1^(3*b*_3*g*_)^1^(1*a*_*u*_)^1^	–2914.555817	7.71	0.208	0.048
^7^ *B*_2*u*_	(10*a*_*g*_)^2^(6*b*_3*u*_)^1^(7*b*_2*u*_)^2^(8*b*_2*u*_)^2^(4*b*_1*g*_)^1^(4*b*_1*u*_)^1^(2*b* ^2*g*^)^1^(3*b*_3*g*_)^1^(1*a*_*u*_)^1^	–2914.568104	0.00	0.142	0.038
^7^ *B*_3*u*_	(10*a*_*g*_)^2^(6*b*_3*u*_)^1^(7*b*_2*u*_)^1^(8*b*_2*u*_)^1^(4*b*_1*g*_)^1^(4*b*_1*u*_)^2^(2*b* ^2*g*^)^1^(3*b*_3*g*_)^1^(1*a*_*u*_)^2^	–2914.531997	22.66	0.301	0.049

a The total CCSD­(T) energies (*E*_total_ in Hartree) and the relative energies (Δ*E* in kcal mol^–1^) between the valence states of Co_2_O_2_. The *D*_1_ and *T*_1_ diagnostics are also reported.

The *T*_1_ ^67^ and *D*_1_ ^68,69^ diagnostics values are used to estimate the multireference (MR) character and quality of the coupled-cluster wave functions. The general criteria for using single reference methods in the calculation of energy levels and spectroscopic properties of molecules containing 3*d* elements are *T*_1_ ≤ 0.05 and *D*_1_ ≤ 0.15.^70^ The *D*_1_ diagnostic values reported in Table 2 are in the range of [0.138, 0.502] suggesting that the valence states have significant MR character, whereas the small *T*_1_ values are in the range of [0.027, 0.085] for the studied valence states, which does not indicate any serious MR problems for most of the states studied. Only three of the 25 valence states have *T*_1_ values greater than the MR threshold of 0.05. The *T*_1_ values are small, probably because the energetically lowest electron configuration obtained in the SA-CASSCF calculations was used as the reference determinant in the CCSD­(T) calculations. Since almost all *D*_1_ diagnostic values exceed the MR threshold, we conclude that the MR character is significant implying that reliable predictions of the relative energy levels require that both static and dynamic correlation effects are considered.

The length of the Co–O bonds optimized at the CCSD­(T) level varies between 1.727 Å for the ^1^ *A*_*g*_ state to 1.859 Å for the ^7^ *B*_3*g*_ state, which can be compared to the experimental value of 1.765 ± 0.01.^40^ The Co–Co distance follows the same trend with the shortest distance for the low-spin states. Bond lengths and bond angles optimized at the CCSD­(T) level are reported in the Supporting Information (SI). The zero-point energy (ZPE) corrections calculated at the PBE level are also reported in the SI. They are generally larger for low-spin states than for high-spin states because the low-spin states have shorter Co–O and Co–Co distances than the high-spin states.

### 3.3 CASSCF and CASPT2 Calculations

CASSCF and CCSD­(T) calculations using the aug-cc-pVTZ and the aug-cc-pVQZ-t basis sets predict that ^7^ *B*_2*u*_ is the ground state. The ^7^ *B*_3*g*_ state is only 0.27 kcal mol^–1^ higher in energy. The ZPE correction calculated at the PBE level for ^7^ *B*_3*g*_ is 2.08 kcal mol^–1^, which is 1.33 kcal mol^–1^ smaller than obtained for the ^7^ *B*_2*u*_ state. It is 1.42 kcal mol^–1^ smaller than the energy of the ^5^ *A*_*g*_ state, and 1.78 kcal mol^–1^ smaller than the energy of the ^3^ *B*_2*u*_ state. The five lowest-lying states at the CASSCF level including ZPE corrections are ^7^ *B*_3*g*_, ^7^ *B*_2*u*_, ^5^ *A*_*g*_, ^3^ *B*_2*u*_, and ^7^ *A*_*u*_, where ^7^ *B*_3*g*_ is the ground state.

CASPT2 calculations that simultaneously consider static and dynamic correlation effects were also performed. Relative energies calculated at the CASSCF and CASPT2 levels are reported in Table 3, where one can see that dynamic correlation effects generally stabilize low-spin states, whereas the relative energy of the high-spin states are shifted upward. Some exceptions are the ^5^ *B*_1*g*_ and ^5^ *B*_2*u*_ states, which are stabilized by dynamic correlation effects, whereas dynamic correlation effects increase the relative energy of the ^3^ *B*_2*u*_ state, which is a low-lying state at the CASSCF level. The same trend is obtained with the triple-ζ quality basis sets. The total energies calculated at the CASSCF/aug-cc-pVTZ and CASPT2/aug-cc-pVTZ levels are reported in the SI. The configuration interaction (CI) coefficient (*C*_0_) and $C_{0}^{2}$ of the leading configuration of the CASSCF wave functions are also given in Table 3. The CASPT2 calculations predict that the ^1^ *A*_*g*_ state is the ground state. The ^3^ *A*_*g*_ and ^5^ *B*_1*g*_ states are 4.35 kcal mol^–1^ and 3.31 kcal mol^–1^ above the ^1^ *A*_*g*_ ground state at the CASPT2/aug-cc-pVQZ-t level. Since the ZPE corrections calculated at the PBE level for ^1^ *A*_*g*_, ^3^ *A*_*g*_, and ^5^ *B*_1*g*_ are 4.82 kcal mol^–1^, 4.35 kcal mol^–1^ and 4.14 kcal mol^–1^, respectively, the ZPE corrections do not alter the order of the lowest states. Since the ^5^ *B*_1*g*_ state is only 3.31 kcal mol^–1^ above the ^1^ *A*_*g*_ ground state, spin–orbit effects might be relevant for their energetic order.

**Table 3:** Relative Energies (In kcal mol^–1^) of Low-Lying Electronic States Calculated at the CASSCF (Δ*E*(CASSCF)) and CASPT2 (Δ*E* (CASPT2)) Levels of Theory Using the Truncated aug-cc-pVQZ Basis Sets (aug-cc-pVQZ-t)^a^

State	Δ*E* (CASSCF)	Δ*E* (CASPT2)	ΔPT2	*C*_0_	$C_{0}^{2}$
^1^ *A*_*g*_	23.69	0.00	–23.69	0.519	0.269
^3^ *A*_*g*_	36.73	4.35	–32.38	0.455	0.207
^3^ *B*_1*u*_	23.78	7.09	–16.69	0.356	0.127
^3^ *B*_2*u*_	4.25	13.86	+9.61	0.340	0.115
^5^ *A*_*g*_	4.26	21.52	+17.26	0.459	0.211
^5^ *B*_1*g*_	30.14	3.31	–26.83	0.459	0.211
^5^ *B*_2*g*_	18.19	15.25	–2.94	0.409	0.168
^5^ *B*_3*g*_	19.03	12.09	–6.94	0.410	0.168
^5^ *B*_1*u*_	12.21	14.09	+1.88	0.299	0.090
^5^ *B*_2*u*_	30.43	12.71	–17.72	0.442	0.195
^7^ *A*_*g*_	26.02	26.45	+0.43	0.634	0.402
^7^ *B*_3*g*_	0.27	26.84	+26.57	0.603	0.364
^7^ *A*_*u*_	9.26	19.48	+10.22	0.614	0.377
^7^ *B*_1*u*_	18.74	31.23	+12.49	0.571	0.326
^7^ *B*_2*u*_	0.00	27.30	+27.30	0.913	0.834

a The differences in the relative energy of the CASSCF and CASPT2 calculations (ΔPT2) are also given . The molecular structures were optimized at the CCSD­(T)/aug-cc-pVTZ Level. The CI coefficients *C*_0_ and $C_{0}^{2}$ of the main configuration of the CASSCF wave function are also reported.

Wave functions exhibit a significant MR character when *C*_0_ ≤ 0.95 or $C_{0}^{2}\leq 0.90$ .^70^ Thus, almost all of the valence states we have studied at the CASSCF level are MR states, which is also consistent with the *D*_1_ diagnostic values calculated at the CCSD­(T) level. The *C*_0_ coefficient of the ^1^ *A*_*g*_ state, which is the ground state at the CASPT2 level, is 0.519 at the CASSCF level, implying that only half of the wave function is described by the reference Slater determinant. The range of *C*_0_ is [0.299–0.913], where the largest *C*_0_ is obtained for the ^7^ *B*_2*u*_ ground state at the CCSD­(T) level of theory. Since the ^7^ *B*_2*u*_ state is dominated by one Slater determinant, it is well described at the CCSD­(T) level and has the smallest *D*_1_ value.

### 3.4 Magnetic Properties

The magnetically induced current density (MICD) of the ^1^ *A*_*g*_ state was calculated using a series of DFT functionals: BP86,^28,29^ PBE,^30^ TPSS,^34^ and the hybrid functionals TPSSh^34,35^ B3LYP^31,32^ PBE0 ^33^ BHandHLYP^71^ and ωB97X^36^ having 10%, 20%, 25%, 50% and range-dependent (22% at short-range, 100% at long-range) Hartree–Fock exchange, and the local hybrid strong-correlation scLH22t^72^ functional. The truncated pcseg-3 (pcseg-3-t) basis set was employed, where the *g* and *h* basis functions of the pcseg-3 basis set were excluded due to limitations in the Turbomole module needed for the input data of the orbital-contribution calculations. The average integrated MIRC strength of the singlet is 24.2 nA/T is almost independent of the functional. The MIRC strengths obtained using various functionals are listed in Table 4. In the analysis, we use the MICD obtained with the TPSSh functional.

**Table 4:** Integrated MIRC Strength ((*I*) in nA/T) of the ^1^ *A*_*g*_ State of Co_2_O_2_ Calculated Using Various DFT Functionals and the pcseg-3-t Basis Set^a^

DFT functional	*I*
BP86	24.6
PBE	24.3
TPSS	24.6
TPSSh	24.6
B3LYP	23.9
PBE0	24.0
BHandHLYP	23.3
ωB97X	24.5
scLH22t	24.3

a The average (*I*) is 24.2 nA/T at the DFT level, and 24.4 nA/T when excluding the MIRC calculated at the BH and HLYP level.

The streamline plot of the MICD of the ^1^ *A*_*g*_ state of Co_2_O_2_ in Figure 2 shows that the MICD consists of a strong diatropic contribution around the molecular ring and local MICD vortices in the vicinity of the atoms. The strength of the magnetically induced ring current (MIRC) was obtained by integrating the MICD passing through a plane perpendicular to the molecular ring. The integration plane begins in the geometric center of the molecule and passes through one of the oxygen atoms. It extends outward to a large distance as shown in Figure S1 of the SI. The MIRC profile was obtained by splitting the integration domain into thin vertical slices that were integrated separately. The MIRC profile in Figure S1 of the SI shows how its strength varies along the integration plane. The net MIRC strength at the TPSSh level is 24.6 nA/T consisting of a strong diatropic MIRC contribution around the molecular ring. There is no paratropic MIRC inside the four-membered ring, which is usually the case for organic molecules that sustain a diatropic MIRC on the outside of the molecular ring and a paratropic ring current inside it.^73^

**Figure 2:**
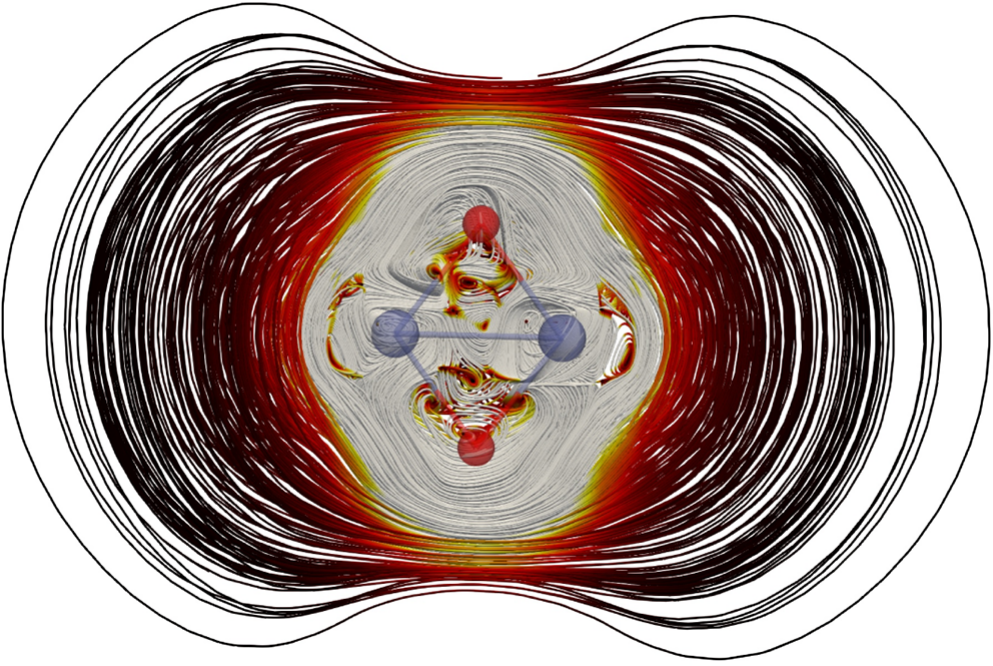
Streamline representation of the MICD of the ^1^ *A*_*g*_ state of Co_2_O_2_ calculated using the TPSSh functional. Oxygen and cobalt are shown in red and blue, respectively. White streamlines are the strongest and the black ones are weakest with red as intermediate strength.

An analysis of the orbital contributions to the MIRC^74^ shows that the diatropic MIRC originates from both σ and π orbitals. The point group of the molecular structure of Co_2_O_2_ in the presence of a magnetic field perpendicular to the molecular ring reduces to *C*_2h_.^75^ The MIRC of all orbitals in each irreducible representation of the *C*_2h_ point group is invariant to the position of the integration plane, whereas the individual orbital contributions are not.^74^ The orbital contributions are obtained as the integrated average of the MIRC strength calculated by rotating the integration plane 360° around the ring in steps of 10°. The MIRC strengths of the orbitals are presented as a histogram in Figure 3. The 10 lowest-lying core orbitals have a negligible contribution to the MICD. Most of the other orbitals have moderate diatropic contributions. The HOMO–6 (*a*_*g*_) and HOMO–3 (*b*_*g*_) orbitals have large paratropic contributions, which are compensated for by the other orbitals, yielding a net diatropic current that is twice as strong as that of benzene.^73^ The strongest diatropic contribution of 14.3 nA/T originates from the *b*_*u*_ orbitals. The combined contributions of σ (*a*_*g*_ and *b*_*u*_) and π (*b*_*g*_ and *a*_*u*_) orbitals are 16.8 nA/T and 7.9 nA/T, respectively. The strong MIRC in the σ orbitals is most likely due to the strain in the four-membered ring and the MIRC contribution of the π orbitals can be assigned to aromatic delocalization. The MIRC strength of all orbitals in each irreducible representation of the *C*_2h_ point group is given in Table 5.

**Table 5:** Contributions to the MIRC Strength (*I* in nA/T) from All Orbitals of the Four Irreducible Representations (Irrep) of the *C*_2h_ Point Group^a^

Irrep	Symmetry	*I*
*a*_g_	*x* ^2^, *y* ^2^, *z* ^2^, *xy*, σ	2.5
*a*_u_	*z*, π	9.7
*b*_g_	*xz*, *yz*, π	–1.9
*b*_u_	*x,y*, σ	14.3

a The symmetry of the irreps with respect to the molecule in the *xy* plane is also given.

**Figure 3:**
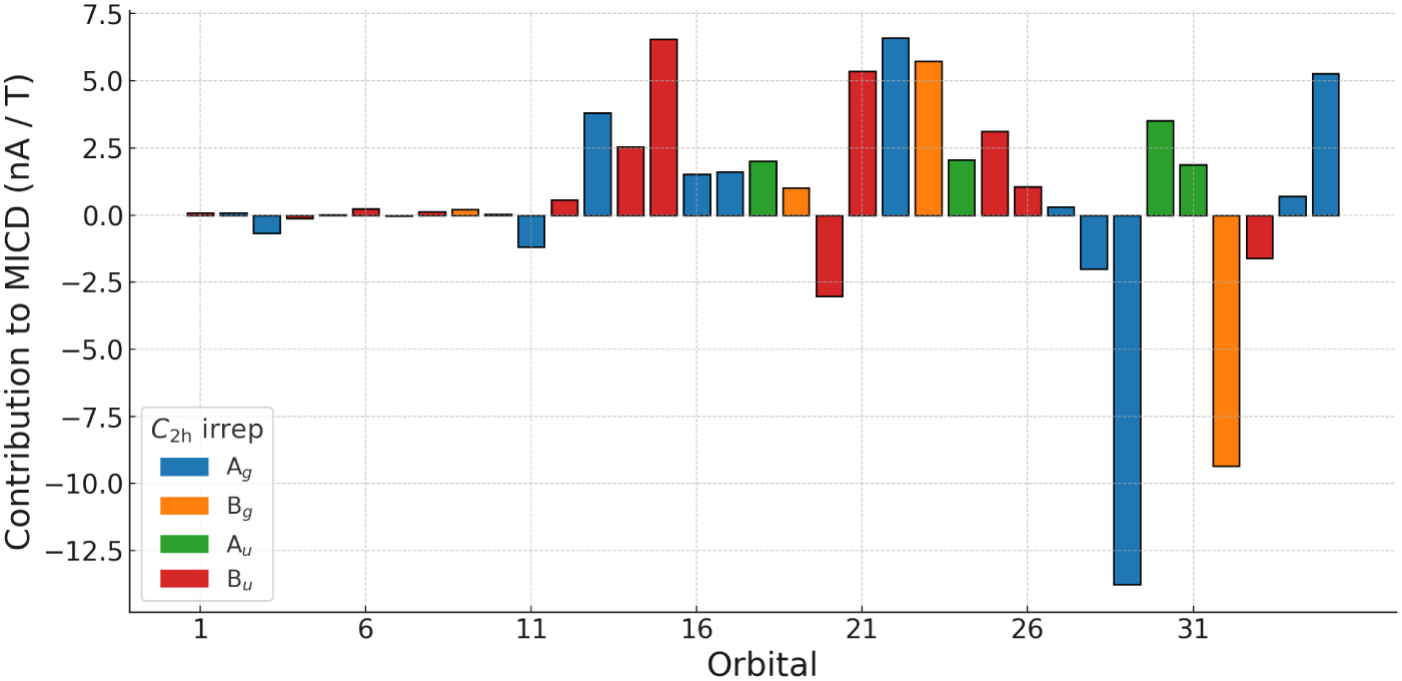
Contributions to the MICD from each orbital. The order on the *x*-axis is according to the orbital energy, i.e., orbital 35 is HOMO. The color scheme represents the irreducible representations of the *C*_2h_ point group.

## 4 Discussion and Conclusions

We have studied the electronic structure of rhombic Co_2_O_2_ at density functional theory (DFT) levels and *ab initio* correlation levels of theory using large basis sets. Calculations were performed at both single-reference and multireference (MR) levels of theory. The DFT calculations suggest that the ground state of Co_2_O_2_ is a high-spin state (*S* = 3). Increasing the amount of Hartree–Fock (HF) exchange in the functional stabilizes high-spin states. The ^5^ *B*_2*g*_ state is the ground state when using the BP86 and PBE functionals at the generalized gradient approximation (GGA), whereas calculations using the meta-GGA functional (TPSS), the hybrid functionals (B3LYP, PBE0, and TPSSh), and the range-separated functional (ωB97X) suggest that the ground state is a septet state. The quintet state is the ground state in calculations at the TPSSh level without any symmetry constraints. The order and the relative energies of the lowest states are not significantly affected by scalar relativistic effects. A low-spin state is the ground state at fully relativistic level of theory without any symmetry constraints. The singlet state is the ground state in broken-symmetry calculations. In the DFT calculations without symmetry constraints and in the broken-symmetry DFT calculations, detailed information about the character of the states is lost because they are not pure states.

Calculations at the singles and doubles coupled-cluster level augmented with a perturbative treatment of the triples (CCSD­(T)) suggest that the ^7^ *B*_2*u*_ state is the ground state and almost degenerate with the ^7^ *A*_*u*_ state. Considering the zero-point energies (ZPE) at the PBE level increases the energy difference between ^7^ *A*_*u*_ and ^7^ *B*_2*u*_. Calculations at the complete active space self-consistent field (CASSCF) level show that the ^7^ *B*_2*u*_ state is completely dominated by one Slater determinant (*C*_0_ = 0.913) and is therefore well described by the single-reference methods. The *C*_0_ coefficient of ^7^ *A*_*u*_ is 0.614, whereas the low-spin and intermediate-spin states have very small *C*_0_ coefficients, suggesting that they cannot be accurately described using single-reference levels of theory.

Calculations at the CASSCF level suggest ^7^ *B*_2*u*_ is the high-spin ground state. Considering static and dynamic electron correlation in the calculations at the second-order complete active space perturbation theory (CASPT2) level changes the order of the states. The ^1^ *A*_*g*_ state is the ground state at the CASPT2 level even though it is 23.69 kcal mol^–1^ above the ^7^ *B*_2*u*_ ground state at the CASSCF and CCSD­(T) levels. The ^5^ *B*_1*g*_ state is the first excited state at the CASPT2 level. It is shifted from 30.14 kcal mol^–1^ above the ^7^ *B*_2*u*_ ground state at the CASSCF level to only 3.31 kcal mol^–1^ above the ^1^ *A*_*g*_ ground state at the CASPT2 level. The ^7^ *B*_2*u*_ state is 27.30 kcal mol^–1^ above the ^1^ *A*_*g*_ ground state at the CASPT2 level. Thus, both static and dynamic correlation effects must be considered to predict the ground state and the correct order of the low-lying states of Co_2_O_2_.

The relative energies obtained at the CCSD­(T), CASSCF, and CASPT2 levels as well as at the DFT level using various functionals are compared in Figure 4, where one can see that single-reference methods incorrectly predict that the ground state is a high-spin state. Even though calculations employing single-reference methods yield consistent results and CASSCF calculations yield a septet ground state, the CASPT2 calculations show that Co_2_O_2_ has a singlet ground state. The CASSCF calculations fail because of missing dynamic electron correlation effects, whereas the CCSD­(T) calculations predict incorrect ground state because most of the valence states of Co_2_O_2_ have significant multireference character, which is also the reason why DFT calculations are inaccurate.

**Figure 4:**
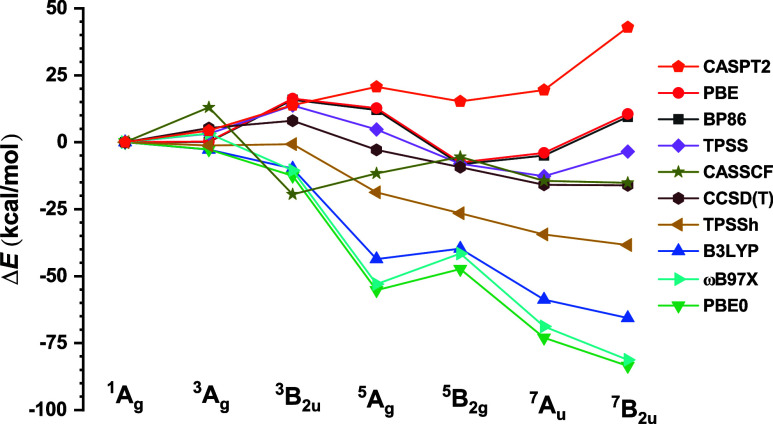
Relative energies of the lowest states calculated at various levels of theory. The aug-cc-pVQZ-t basis sets were used in CASSCF and CASPT2 calculations, and aug-cc-pVTZ basis sets were used for all other levels of theory. The energy of ^1^ *A*_*g*_ is set to zero as the reference.

The lowest states are almost degenerate at some levels of theory implying that zero-point energy corrections can change the order of them. Relativistic effects including spin–orbit coupling have not been considered. The spin–orbit coupling can introduce uncertainties in the order of the states when they are almost degenerate. The spin–orbit splitting of the ground state of Co^2+^ is less than 2.5 kcal mol^–1^, which is probably an upper bound for the spin–orbit splitting of low-lying states of Co_2_O_2_ because the experimental spin–orbit coupling constant of CoO is only 163 cm^–1^.^18^

DFT calculations of the magnetically induced current density (MICD) susceptibility of the ^1^ *A*_*g*_ state of Co_2_O_2_ show that the four-membered ring sustains a net diatropic magnetically induced ring current (MIRC) of 24.6 nA/T, originating from both σ and π orbitals. The MIRC in the σ orbitals of 16.8 nA/T is most likely due to the ring strain, whereas the π contribution of 7.8 nA/T can be assigned to aromatic delocalization.

## Supplementary Material





## Data Availability

The data supporting the results reported in this article have been included as part of the SI. The webpages of MOLPRO and Turbomole are www.molpro.net/ and www.turbomole.org/, respectively. GIMIC, version 2.0 can be freely downloaded from github.com/qmcurrents/gimic and zenodo.org/record/8180435.

## Notes

The SI contains total and relative energies calculated at the CASSCF and CASPT2 levels, the largest CI coefficient of the CASSCF calculations, the molecular structures optimized at the CCSD­(T) and CASSCF levels, zero-point energies calculated at the PBE level, and a picture of the MIRC profile (PDF)
The coordinate.zip file contains the coordinates of the optimized molecular structures (ZIP)
