# Supplementary material for: The Electronic Structure of Planar Rhombic Co2O2
Source: J Phys Chem A. 2026 Jan 12;130(3):614–22. doi: 10.1021/acs.jpca.5c06695 (PMC12833863; doi:10.1021/acs.jpca.5c06695)
Supplement: Supplementary file 1 [file jp5c06695_si_001.pdf]

Supporting Information

The Electronic Structure of Planar Rhombic Co_2O_2

Dou Du,[†] Namin Xiao,[‡] Xingwu Li,[‡] Maria Dimitrova,[*,¶] Dage Sundholm,[*,¶] and Xiao-Gen Xiong[*,§]

†*Materials Evaluation Center for Aeronautical and Aeroengine Application, AECC Beijing Institute of Aeronautical Materials, Beijing 100095, China.*

‡*AECC Beijing Institute of Aeronautical Materials, Beijing 100095, China.*

¶*Department of Chemistry, Faculty of Science, University of Helsinki, P.O. Box 55, A. I. Virtasen aukio 1, FIN-00014 Helsinki, Finland*

§*Sino-French Institute of Nuclear Engineering and Technology, Sun Yat-sen University, Zhuhai 519082, P. R. China. and CNPRI-SYSU Joint Research Center for Coolant Chemistry of Nuclear Reactor, Zhuhai 519082, P. R. China.*

E-mail: maria.dimitrova@helsinki.fi; dage.sundholm@helsinki.fi; xiongxg@mail.sysu.edu.cn

Table S1: The total energies (in hartree) calculated at the CASSCF and CASPT2 levels. The energy differences (in kcal mol^{-1}) relative to the ground state are also given. The CCSD(T)/aug-cc-pVTZ optimized geometries and aug-cc-pVTZ basis sets were used in the CASSCF and CASPT2 calculations.

State	E(CASSCF)	ΔE(CASSCF)	E(CASPT2)	ΔE(CASPT2)	C_0	C_0^2
^1A_g	-2912.622722	23.75	-2914.514382	0.00	0.533	0.284
^3A_g	-2912.602089	36.70	-2914.509974	2.77	0.484	0.234
$^3B_{1u}$	-2912.622736	23.74	-2914.505607	5.51	0.399	0.159
$^3B_{2u}$	-2912.651931	5.42	-2914.492120	13.97	0.358	0.128
^5A_g	-2912.655222	3.36	-2914.485180	18.32	0.476	0.227
$^5B_{2g}$	-2912.631632	18.16	-2914.496249	11.38	0.475	0.226
$^5B_{3g}$	-2912.630283	19.01	-2914.497072	10.86	0.460	0.212
$^5B_{1u}$	-2912.641188	12.16	-2914.492819	13.53	0.376	0.141
$^5B_{2u}$	-2912.612119	30.40	-2914.497025	10.89	0.477	0.228
^7A_g	-2912.619139	26.00	-2914.479493	21.89	0.712	0.507
$^7B_{3g}$	-2912.660199	0.23	-2914.477809	22.95	0.697	0.486
^7A_u	-2912.645867	9.23	-2914.489076	15.88	0.755	0.570
$^7B_{1u}$	-2912.635799	15.54	-2914.480835	21.05	0.761	0.579
$^7B_{2u}$	-2912.660571	0.00	-2914.474215	25.20	0.970	0.940

Table S2: The total energies (in hartree) calculated at the CASSCF and CASPT2 levels. The CCSD(T)/aug-cc-pVTZ optimized geometries and aug-cc-pVQZ-t basis sets were used in the CASSCF and CASPT2 calculations. The highest-angular-momentum functions of the aug-cc-pVQZ basis sets were removed. The CI coefficient C_0 and weight C_0^2 of the leading configuration in the CASSCF wave function are also included.

State	E(CASSCF)	E(CASPT2)	C_0	C_0^2
^1A_g	-2912.634323	-2914.682676	0.519	0.269
^3A_g	-2912.613537	-2914.675738	0.455	0.207
$^3B_{1u}$	-2912.634166	-2914.671372	0.356	0.127
$^3B_{2u}$	-2912.665304	-2914.660583	0.340	0.115
^5A_g	-2912.665279	-2914.648379	0.459	0.211
$^5B_{1g}$	-2912.624043	-2914.677408	0.459	0.211
$^5B_{2g}$	-2912.643075	-2914.658367	0.409	0.168
$^5B_{3g}$	-2912.641739	-2914.663403	0.410	0.168
$^5B_{1u}$	-2912.652617	-2914.660215	0.299	0.090
$^5B_{2u}$	-2912.623575	-2914.662427	0.442	0.195
^7A_g	-2912.630596	-2914.640526	0.634	0.402
$^7B_{3g}$	-2912.671631	-2914.639899	0.603	0.364
^7A_u	-2912.657313	-2914.651640	0.614	0.377
$^7B_{1u}$	-2912.642202	-2914.632903	0.571	0.326
$^7B_{2u}$	-2912.672069	-2914.639169	0.913	0.834

Table S3: Equilibrium bond lengths and angles of Co_2O_2 obtained at the CCSD(T)/aug-cc-pVTZ level. All bond lengths are in Å and all angles are in degree. The zero point energies (ZPE in kcal mol^{-1}) calculated at the PBE/def2-TZVPP level are also reported.

State	R(Co-O)	R(Co-Co)	∠Co-O-Co	ZPE
^1A_g	1.727, 1.876[a]	2.048	72.75, 81.17[a]	4.82
^3A_g	1.747, 1.894[a]	2.095	73.71, 80.10[a]	4.35
$^3B_{1u}$	1.747, 1.878[a]	2.070	72.67, 81.14[a]	4.47
$^3B_{2u}$	1.781	2.279	79.56	3.86
^5A_g	1.797	2.294	79.33	3.50
$^5B_{1g}$	1.761, 1.890[a]	2.066	71.84, 81.40[a]	4.15
$^5B_{2g}$	1.768	2.197	76.84	4.05
$^5B_{3g}$	1.779	2.092	72.03	3.12
$^5B_{1u}$	1.776	2.216	77.22	3.44
$^5B_{2u}$	1.776	2.121	73.34	2.78
^7A_g	1.822	2.133	71.63	3.06
$^7B_{3g}$	1.859	2.447	82.31	2.08
^7A_u	1.811, 1.889[a]	2.252	76.90, 81.43[a]	3.74
$^7B_{1u}$	1.826	2.110	70.59	3.10
$^7B_{2u}$	1.832	2.369	80.56	3.41
Exp.[b]	1.765±0.01		87±5	

[a] Optimized at the CASSCF/aug-cc-pVTZ level

[b] Danset, D.; Manceron, L. *Phys. Chem. Chem. Phys.* **2005**, *7*, 583–591

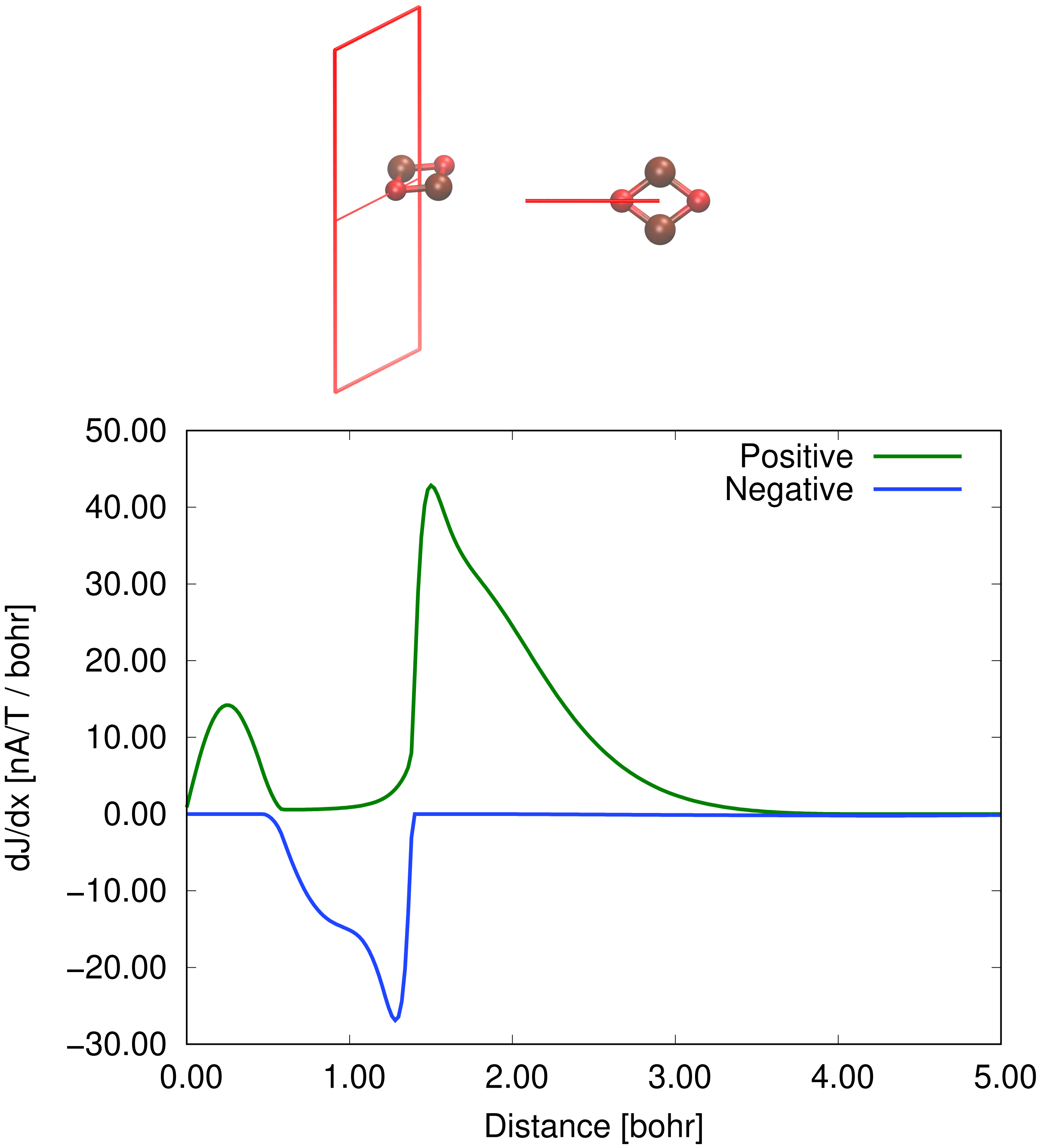

Figure S1: The upper figure shows the integration plane placed between the cobalt atoms (in brown) that passes one of the oxygen atoms (in red). The MIRC profile calculated at the TPSSh/pcseg-3-t level shows the strength of the MICD passing through the integration plane. The positive green profile shows the diatropic contribution and the blue profile originates from the local diatropic MICD vortex around the oxygen atom.
